# Supplementary material for: Development and Cell Cycle Activity of the Root Apical Meristem in the Fern Ceratopteris richardii
Source: Genes (Basel). 2020 Dec 4;11(12):1455. doi: 10.3390/genes11121455 (PMC7761924; doi:10.3390/genes11121455)
Supplement: Supplementary file 1 [file genes-11-01455-s001.pdf]

Supplementary Materials

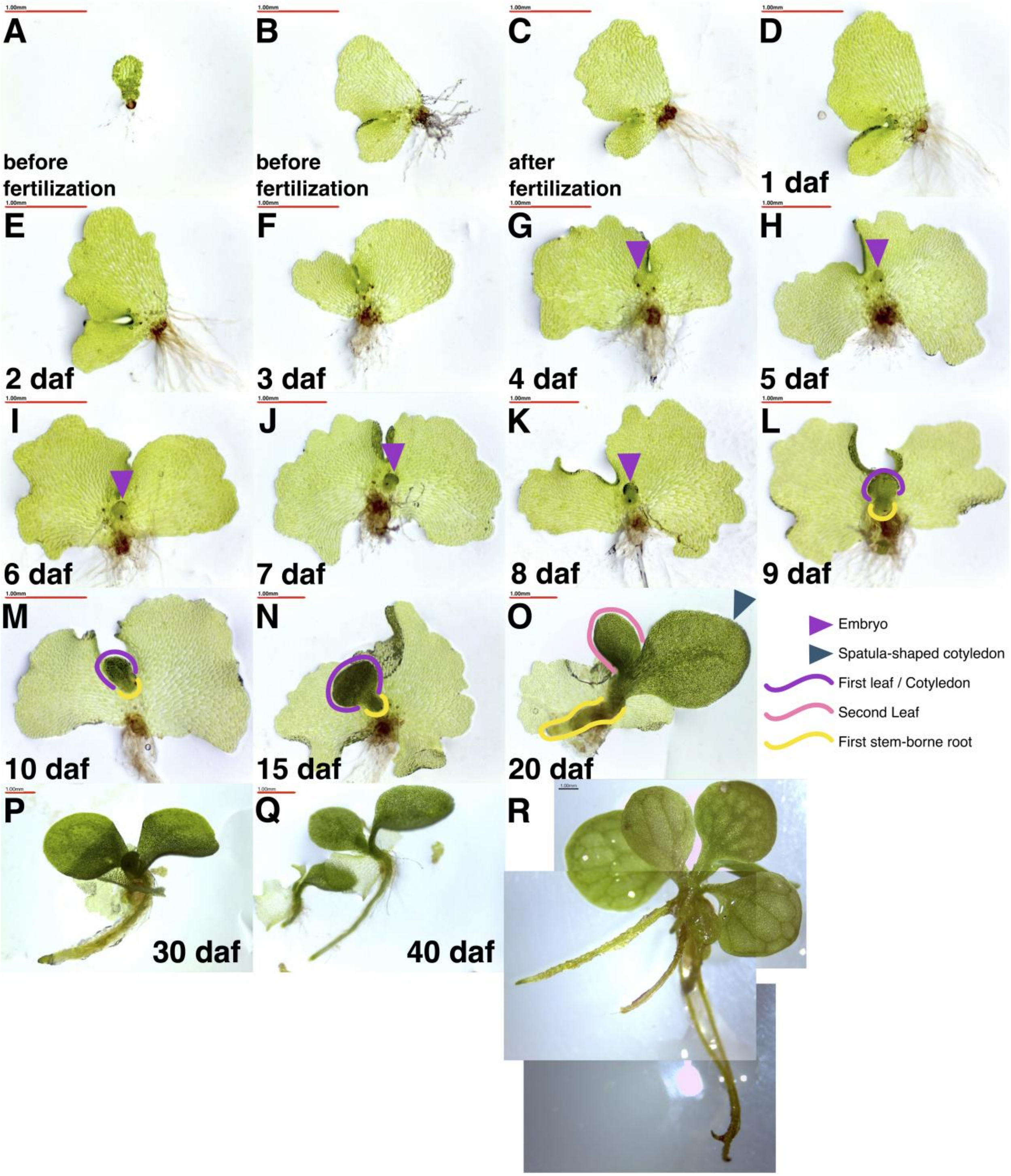

Figure S1. Bright-field images of gametophyte-to-sporophyte transition and embryo development in *Ceratopteris richardii*. (A) Male gametophyte at 15 dps. (B to O) Transitions of a hermaphrodite gametophyte from reaching its sexual maturity at 15 dps through fertilization, embryo development and young sporophyte development.

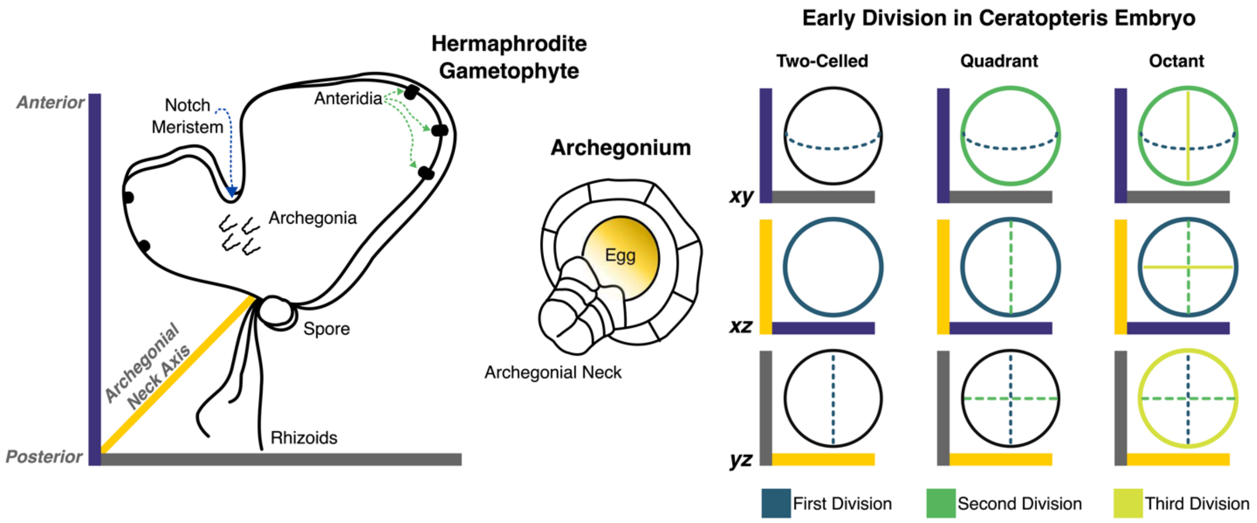

Figure S2. Graphic representation of different planes in a hermaphrodite gametophyte. The anterior-posterior axis is adjusted from the notch meristem to the rhizoids, respectively. Archegonia are localized below the notch meristem; each archegonium contains an egg. After fertilization, the one-celled zygote divides continuously to promote further development. The different cell walls from these early divisions can be distinguished according to which position the embryo is observed.

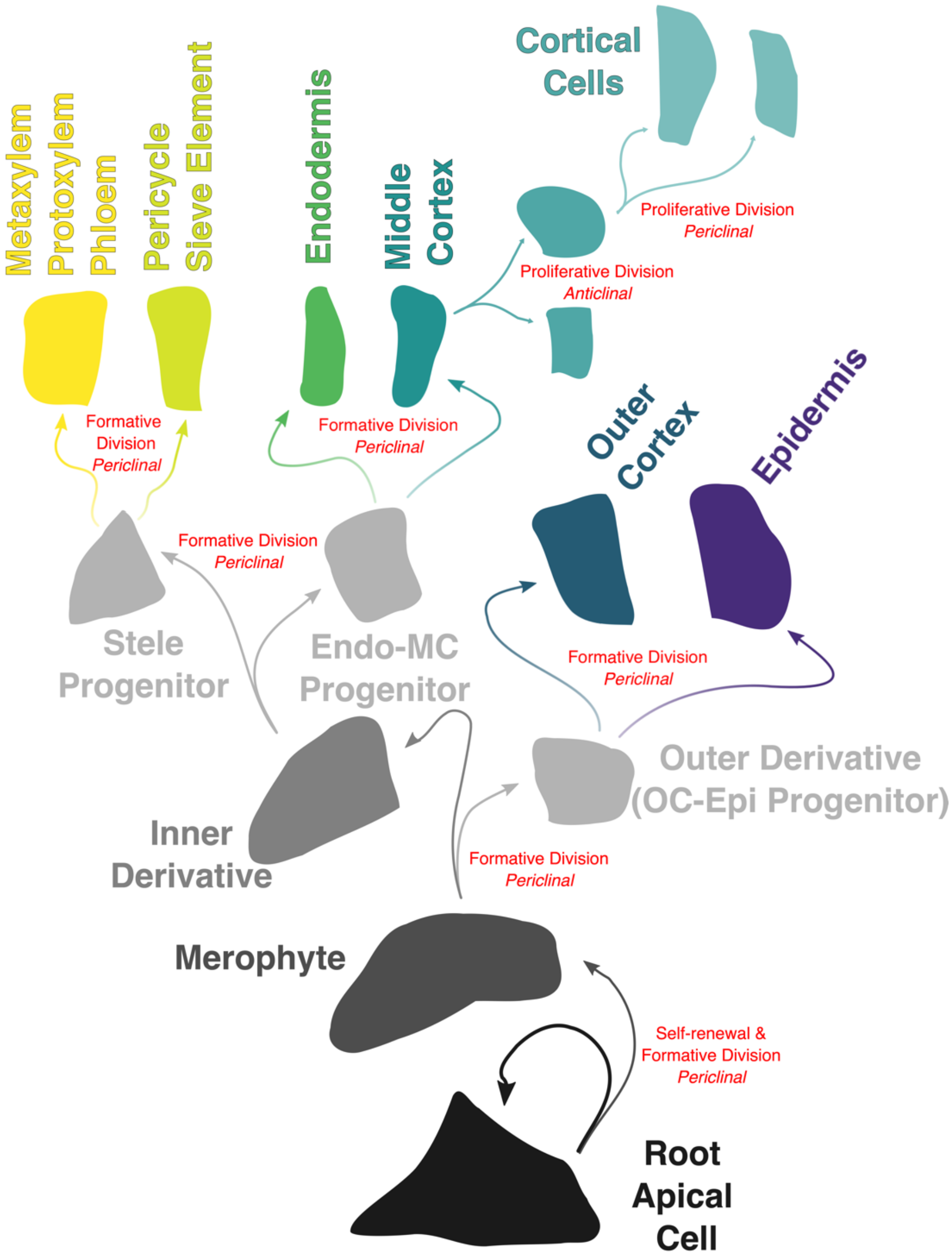

Figure S3. Schematic representation of cell ontogeny in the root body. The RAC divides constantly renewing itself and generating new merophytes. Each proximal merophyte will generate all different cell fates through a series of formative asymmetric divisions. The first divisions produce three types of derivatives: stele derivative (inner derivative), endodermis & middle cortex derivative (middle derivative), outer cortex & epidermis derivative (outer derivative). These cells divide later to generate daughters forming the diverse cell layers. Proliferative divisions can occur at this stage to expand cell number in each layer; for example, middle cortex cells divide anticlinally and periclinally to further differentiate in cortical aerenchyma. In a previous analysis, derivative cells were shown to undergo proliferative divisions during their development (Hou and Hill, 2004), but these divisions are not trackable using longitudinal planes because they are considered radial anticlinal divisions. Epi – Epidermis; Endo – Endodermis; MC – Middle Cortex; OC – Outer Cortex.

Root Meristem Diversity (Figure 1)			
Species	Family	Clade	Reference
Arabidopsis thaliana	Brassicaceae	Angiosperms	[20]
Solanum lycopersicum	Solanaceae	Angiosperms	[90]
Oryza sativa	Poaceae	Angiosperms	[91]
Amborella trichopoda	Amborellaceae	Angiosperms	[21], [51]
Gingko biloba	Ginkgoaceae	Gymnosperms	[51], [92]
Pinus taeda	Pinaceae	Gymnosperms	[93]
Ceratopteris richardii	Pteridaceae	Ferns	[23]
Azolla filiculloides	Salviniaceae	Ferns	[42]
Equisetum diffusum	Equisetaceae	Ferns	[94]
Selaginella kraussiana	Selaginellaceae	Lycophytes	[55]
Isoetes pseudojaponica	Isoetaceae	Lycophytes	[25]
Lycopodium clavatum	Lycopodiaceae	Lycophytes	[25], [51]
Huperzia serrata	Lycopodiaceae	Lycophytes	[25], [58]

Root Meristem Diversity (extended species list)			
Species	Family	Clade	Reference
Several families		Angiosperms	[21], [51]
Stenocereus gummosus	Cactaceae	Angiosperms	[84]
Pachycereus pringlei	Cactaceae	Angiosperms	[84]
Hordeum vulgare	Poaceae	Angiosperms	[44]
Cycas revoluta	Cycadaceae	Gymnosperms	[92]
Ephedra foliata	Ephedraceae	Gymnosperms	[95], [96]
Ephedra sinica	Ephedraceae	Gymnosperms	[51]
Gnetum gnemon	Gnetaceae	Gymnosperms	[51]
Pinus densiflora	Pinaceae	Gymnosperms	[51]
Pseudotsuga taxifolia	Pinaceae	Gymnosperms	[97]
Picea glauca	Pinaceae	Gymnosperms	[98]
Radix carbonica	NA (fossil)	Progymnosperm	[99]
Several families		Ferns	[100]
Platycerium bifurcatum	Polypodiaceae	Ferns	[101]
Hypolepis punctata	Dennstaedtiaceae	Ferns	[25], [51]
Regnellidium diphyllum	Marsileaceae	Ferns	[70]
Osmunda regalis	Osmundaceae	Ferns	[52]
Angiopteris lygodiifolia	Marattiaceae	Ferns	[51]
Equisetum limosum	Equisetaceae	Ferns	[51]

Selaginella uncinata	Selaginellaceae	Lycophytes	[25]
Diphasiastrum complanatum	Lycopodiaceae	Lycophytes	[25]
Lycopodiella cernua	Lycopodiaceae	Lycophytes	[25]

Table S1. Vascular plant species analyzed to generate Fig. 1A. Also including its corresponding reference (upper table). Other plant species taken into account for comparison (lower table).

Growth Rate (mm/day)	Day 0-5	Day 5-10	Day 10-15	Day 15-20	Day 20-25	Day 25-30
SBR-1	0.17	0.69	0.99	0.89	0.61	0.07
SBR-2	A[NS]	B**	0.29/C**	0.98	1.73	1.13
SBR-3	A[NS]	B**	C**	0.25	2.13	1.27
SBR-4				0.02	1.47	1.12
SBR-5					1.01	1.02
SBR-6					0.39	1.31
SBR-7						0.86

Table S2. Root growth rate (RGR) for each stem-borne root according to a five-day timespan. A – comparison of RGR after the first 5 days of active growth of each root; B – comparison of RGR at 10 days of growth; C – comparison of RGR at 15 days of growth. NS – no significant; * - p<0.05; ** - p<0.01.
